# Can Outcomes of a Chat-Based Suicide Prevention Helpline Be Improved by Training Counselors in Motivational Interviewing? A Non-randomized Controlled Trial

**DOI:** 10.3389/fdgth.2022.871841

**Published:** 2022-06-21

**Authors:** Wilco Janssen, Jeroen van Raak, Yannick van der Lucht, Wouter van Ballegooijen, Saskia Mérelle

**Affiliations:** ^1^113 Suicide Prevention, Amsterdam, Netherlands; ^2^Department of Clinical, Neuro and Developmental Psychology, Faculty of Behavioural and Movement Sciences, Vrije Universiteit Amsterdam, Amsterdam, Netherlands; ^3^Department of Psychiatry, Amsterdam UMC, Amsterdam, Netherlands

**Keywords:** motivational interviewing (MI), suicide prevention, helpline, treatment integrity, training, chat

## Abstract

**Objective:**

To examine whether the outcomes of a chat-based suicide-prevention helpline could be improved by training counselors in motivational interviewing (MI).

**Methods:**

In a pre- and post-test design, visitors of a chat-based suicide prevention helpline received either the Five-Phase Model (treatment as usual [TAU]) or MI. They completed a pre- and post-chat questionnaire on several suicide-related risk factors. Linear mixed modeling was used to estimate the effect of the condition. Furthermore, the treatment proficiency of newly trained counselors was assessed using MI-Scope.

**Results:**

A total of 756 visitors and 55 counselors were included in this study. The visitors showed an improvement in suicidal ideation and psychological risk factors after a chat conversation. However, there were no significant differences between the MI and TAU conditions (β = 0.03, 95% CI [−0.23–0.30], *p* = 0.80). The treatment integrity indices showed that the counselors mostly used MI-consistent techniques but were unable to strategically employ these techniques to evoke enough change talk.

**Conclusions:**

MI and TAU led to comparable outcomes in a chat-based suicide prevention helpline. The effectiveness of MI might improve by intensifying or improving the training of counselors, keeping the process of engaging more concise or offering visitors multiple sessions of MI.

## Introduction

Practical and psychological barriers, such as negative attitudes toward help-seeking, stigma and the fear of involuntary hospitalization, make it difficult for people with suicidal thoughts or behavior to seek help (1). Furthermore, suicidal ideation varies in intensity (2), and the transition from suicidal ideation to a suicide attempt can happen within days, hours or even minutes (3). Therefore, several suicide prevention centers offer helplines where people with suicidal thoughts or behavior can get help 24/7, anonymously and free of charge. Indeed, these helplines seem to reach high-risk populations, with the number of suicide attempts among helpline users being more than twice as high as in the general population (4).

Research has consistently shown that suicide helplines can reduce psychological pain, hopelessness and suicidal ideation in callers, although high-quality evidence remains sparse: ethical considerations limit the use of adequate control groups, and the vast majority of studies have relied exclusively on observer ratings, many of which were unvalidated (5). Effect sizes are generally small, and non-response is common. For instance, Mishara et al. (6) found that suicidal urgency decreased in only 16% of users, while 76% showed no change, and 8% seemed to have deteriorated during the conversation.

To date, only two studies have tested the effectiveness of chat-based helplines and found a positive effect on visitor hopelessness and suicidal ideation, among others (7, 8). Gould et al. (8) reported that 45% of the visitors felt less suicidal after the chat. However, approximately 30% reported no change, and 12% deteriorated during their contact with the helpline, while the effect on the remaining callers was unclear.

Mokkenstorm et al. (7) suggested that training counselors in therapeutic techniques might improve the outcomes of suicide prevention helplines. Support for this idea emerged from a randomized controlled trial by Gould et al. (9), who provided Applied Suicide Intervention Skills Training (ASIST) to 764 counselors of a phone-based suicide helpline in the United States and compared their results with those relating to 646 counselors from the same helpline who had not received this training. The group of counselors trained in ASIST showed larger reductions in suicidality, hopelessness and overwhelming feelings than their untrained counterparts. These results support the idea that the effectiveness of helplines may be improved by training counselors in a specific methodology.

Motivational interviewing (MI) seems particularly well suited to suicide prevention helplines (10). MI is a counseling style designed to help people change their behavior by resolving their ambivalence about it. This is important since ambivalence is typical in suicidal behavior [e.g., (11)] and has previously been found to be associated with suicide attempts and death by suicide (12). A large body of evidence shows that MI is effective in reducing several forms of destructive behavior in a wide range of populations (13, 14), and preliminary evidence suggests that MI can also be delivered online [e.g., (15)].

MI has never been applied in a helpline, but there is some evidence to suggest that it can be used for suicide prevention. Two RCTs found that MI was effective at motivating people with suicidal behavior to engage in life-sustaining behavior, such as engaging in mental health care or using a safety plan (16, 17). Furthermore, a pilot study by Britton et al. (18) found that MI could also be used to directly influence the motivation for suicide. A randomized controlled trial by the same authors (19) found no added value in one or two sessions of MI over treatment as usual (TAU) in 132 veterans with suicidal ideation. Their results showed that subjects who received MI were 41% less likely to report suicidal ideation at 6 months follow-up than those who did not, but the difference was not significant. However, the authors noted that their study might have been underpowered and that TAU consisted of an intensive inpatient treatment, which included pharmacotherapy, family counseling and safety planning.

MI can be learned by professionals, volunteers and students alike (14), all of whom are regularly found among helpline staff. However, studies employing fidelity measures have also showed that counselors often failed to reach the beginner proficiency level in MI (20), as defined by Moyers et al. (21). It must be noted, however, that these criteria are based on expert opinion rather than empirical observation and might be overly stringent, given the fact that many studies have failed to meet them, and favorable outcomes have been achieved in studies reporting sub-standard adherence scores (22). These findings warrant further research, particularly in specifying counsellors' training needs in order to adequately deliver MI (23).

The aim of this study was twofold. First, we trained counselors of a chat-based suicide prevention helpline in MI and assessed their proficiency level according to the empirically derived benchmark criteria provided by Fischer (22). Second, we asked visitors to rate themselves on several well-established risk factors for suicide before and after chatting with the helpline and compared the outcomes of chats in which the counselor used the helpline's usual method, called the Five-Phase Model (TAU), with those of chats by the same counselors after being trained in MI. This was therefore a quasi-experimental study. We hypothesize that counselors can reach sufficient proficiency in MI and that visitors treated with MI will benefit more from their chat than those receiving TAU. We expect to find a small effect size.

## Materials and Methods

### Sample

The participants were recruited among people who contacted the 113 Suicide Prevention crisis chat service in the Netherlands between 08:30 AM and 10:30 PM. Visitors were eligible for participation if they spoke Dutch, filled out both the pre- and post-chat questionnaire and reported at least some suicidal ideation on the pre-chat questionnaire (score ≥ 1 on a 7-point scale). Chats were included if they lasted at least 20 min, if triage was not included, and if the attending counselor conducted at least one chat in both conditions.

### Interventions

#### Treatment as Usual

Crisis chats on the 113 Suicide Prevention helpline are usually carried out according to the so-called Five-Phase Model, which was developed by the Dutch Child Helpline to structure online conversations and was found to improve visitor satisfaction (24). The Five-Phase Model is based on Egan's (25) five stages of counseling (2013) and instructs counselors to (1) establish rapport with visitors; (2) clarify the visitor's story; (3) set a goal; (4) work the goal out; and (5) close the conversation.

#### Motivational Interviewing

MI is a counseling style designed to help people alter their behavior. MI can be broadly divided into two components. First, it uses a so-called Rogerian or client-centered conversation style in which the counselor uses non-directive conversation techniques to establish rapport with the visitor and negotiate one or more topics to talk about. The therapist tries to focus on what matters most to the client and is careful not to put any pressure on them. This is the relational component of MI, which helps in avoiding discord and minimizing the triggering of defensive reactions. It is present throughout the conversation but is most prominent during the first two processes of MI: engaging and focusing. The last two processes—evoking and planning—constitute the technical components of MI. The technical component of MI is built on the assumption that people generally have conflicting thoughts and feelings about important decisions, such as the decision to seek mental health treatment, tell their loved ones about their problems or end their own life. This ambivalence makes it difficult for them to reach a decision. Whenever they lean toward one option, the alternative suddenly seems more appealing and vice versa. At the same time, this means that people always have reasons to do something, even when they are not motivated to do so. It may be, for example, that someone does not seek help because they are fearful of involuntary hospitalization but, at the same time, believes that treatment might be helpful. During evoking, people are encouraged to talk about these reasons as much and as vividly as possible, thereby increasing their intrinsic motivation for the target behavior. If the target behavior is to motivate the visitor to seek professional help, for example, the counselor might ask what they think would improve if treatment was successful. If the visitor seems sufficiently motivated to engage in the target behavior, the counselor continues to the process of planning, that is, where the visitor is encouraged to think of concrete steps they might take toward the target behavior (e.g., ask the GP for a referral) and express their commitment to this plan.

MI thus includes all the stages of the Five-Phase model, except for 'clarifying the visitors story'. It differs from the Five-Phase model in that it instructs counselors to use non-directive conversation techniques and gives counselors more guidance on the way in which they are to work out the goal they've agreed upon with the visitor, namely by eliciting change talk and minimizing sustain talk. Research has consistently shown that this is the active ingredient of MI, to which it owes its effectiveness (26).

### Training

#### Treatment as Usual

At the start of their career at the helpline, all counselors had attended a clinical workshop of 32 h on the Five-Phase Model by a licensed clinician, in which they were also taught basic knowledge about suicidal behavior. They also received supervision from a more experienced colleague once every 2–4 weeks.

#### Motivational Interviewing

All counselors of the helpline were invited by e-mail to participate in the study, together with a short description of the time and effort this would require from them. All interested counselors then participated in two clinical workshops on MI, each given by one or two licensed clinicians with extensive experience in MI, suicide prevention and teaching (a more specific description of the contents of the training can be found in Appendix A). Together, the two workshops lasted ~7 h, after which all the counselors could attend weekly coaching sessions led by one of the trainers and were divided into groups of three or four to discuss their performance among peers. In addition, after the second workshop, all counselors were given feedback on at least one chat they conducted and were provided with a recording of both workshops, a handout of the presentation, a workbook and a placemat containing example questions. Apart from these resources, no standardized tools were used during the training or implementation of MI.

### Design

**Figure 1:**
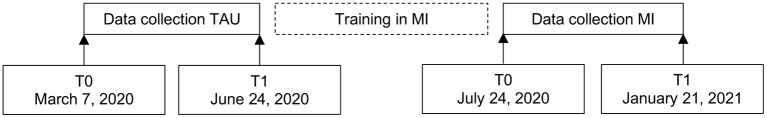
Study design and timeline.

The study used a quasi-experimental design (Figure 1). First, the pre- and post-measurements were collected from visitors receiving TAU. The data collection was then paused, and the participating counselors were trained in MI. The data collection then resumed, continuing until enough data had been collected and every participating counselor conducted at least one chat in the experimental condition. As the two conditions were sampled sequentially, we controlled for the working experience of the participating counselors. Visitors were not aware of the type of treatment they were given. Ethical approval for this study was obtained from the ethics review committee of the VU University Medical Center in Amsterdam (2020.105).

### Procedure

Visitors started the chat by clicking the “chat with us” button on the helpline's website, after which all visitors were presented with the questionnaire in Table 1. This is part of the helpline's standard procedure and is mandatory for all visitors. After filling out the questionnaire, visitors entered a live chat with a triage, who checked whether the situation was safe enough to start a conversation. Visitors who could not be persuaded to keep some distance from lethal means for the duration of the conversation were referred to emergency services, as were people who were seriously injured. The remaining participants were then connected to the first available counselor and received either TAU or MI. During the chat, counselors could consult a senior colleague at any time if they required assistance. The planned duration of a chat was 45 min. After the chat, participants were asked to re-fill the questionnaire in Table 1. Since this was a service evaluation study that did not involve allocation to conditions, the participants were not asked for informed consent.

**Table 1:** Items measuring suicide risk factors.

**Variable**	**Item**	**Source**
Suicidal ideation	I feel the urge to kill myself	(2 )
Unbearable psychache	I can't take my pain anymore	(27)
Hopelessness	I feel hopeless	(28)
Defeat	I feel that I have given up	(29)
Entrapment	I feel trapped	(30)
Perceived burdensomeness	I am a burden to others	(28)
Thwarted belongingness	I feel like I do not belong	(31)
Desire to live	I have the desire to live	(28)
Capability for suicide	I could kill myself if I wanted to	(32)

### Measures

#### Demographics

Self-reported sex and age information was registered.

#### Suicide Risk Factors

The primary outcome variables in this study were chosen on the basis of the prevailing theories on the origins of suicidal behavior: the interpersonal theory (33), the Integrated Motivational Volitional Model (34) and the Three-Step Theory of Suicide (35). Suicide risk factors were measured with items from several ecological momentary assessment studies which also sought to capture changes over a very short time span and were designed to minimize the burden they place on participants. The items were translated into Dutch by the first author, except for the Dutch items (28), and were slightly rephrased where necessary to improve readability. Since none of these studies contained items measuring defeat, one item was selected from the Defeat Scale (36) based on their factor loadings found by Forkmann et al. (37). Items measuring unbearable psychache were drawn from the UP3 (27). All items were measured on a 7-point Likert scale from 1 “completely disagree” to 7 “completely agree”. Table 1 shows the questions posed to the participants before and after each chat.

#### Counselors Working Experience

Working experience was estimated for each counselor by calculating the number of hours the attending counselor had worked for the helpline at the time of the chat. This was done by calculating the difference between the date of the chat and the date on which the counselor had joined the helpline, which was then multiplied by the counsellors' weekly working hours.

#### Proficiency Level in MI

To assess the counsellors' proficiency level, all chats in which MI was used were coded in ATLAS.ti 9 for Windows by two of the authors using a standardized coding system. Following recommendations by Mokkenstorm et al. (7), both the behavior of the counselor and the reactions by the visitor were coded. This study used the MI-SCOPE (38), which covers more aspects of MI than other instruments and is more time-efficient than some of the other instruments (39). Five indices of treatment integrity can be extracted from the MI-SCOPE: the percentage of MI-consistent responses, the percentage of open questions, the percentage of complex reflections, the reflection-to-question ratio and the proportion of change talk. A review by Hurlocker et al. (40) showed that reliability estimates for the MI-SCOPE are generally fair to excellent.

### Statistical Methods

All analyses were conducted in R-Studio, Version 1.10.1093. Chi-square tests and independent t-tests were used to test group differences at baseline. Linear mixed models were used to investigate changes between the two conditions across time on suicidal ideation, unbearable psychache, hopelessness, defeat, entrapment, perceived burdensomeness, thwarted belongingness, reasons for living and capability for suicide. All models were extended in a stepwise manner for each variable and compared. The null model consisted of the fixed effect of time, condition and time × condition, with the counselor who handled the chat added as a random effect to account for the fact that visitors were nested within counselors. The counsellor's working experience was then added.

All outcome variables were measured on a 7-point Likert scale. Since skewedness toward the high end of these scales was observed, the variances were not normally distributed. Therefore, the linearity assumption of the Likert scales was further investigated by estimating an ordered logistic regression model. Since there were no large differences in the threshold intervals, we assumed that this distribution most likely did not affect the mixed-model analysis.

#### Coding Procedure

Following recommendations by O'Connor and Joffe (41), the first chats were coded by two research assistants (the second and third authors). They double-coded random chats from the helpline to learn how to use the MI-SCOPE and refine the coding frame under the supervision of the first author. Afterwards, 10% of the chats in the MI condition were selected, and both coders independently coded half of these chats. Both coders could consult with the first author if they were in doubt about the appropriate code. After intercoder reliability was found to be sufficient (see section Inter-coder Reliability), the remaining chats were coded following the same procedure.

#### Inter-coder Reliability

Following recommendations from Hallgren (42), inter-coder reliability was computed over the MI-SCOPE summary scores in their final transformed form—not over each code separately—as only the summary scores were used for the analyses. Intercoder reliability was assessed by computing Krippendorff's (43) alpha-binary using Atlas.ti 9, a method suitable for two or more coders, which can incorporate all data types. Furthermore, there is general agreement on how the results are to be interpreted: an alpha-binary over 0.9 is always acceptable; an alpha-binary between 0.8 and 0.9 is generally regarded as sufficient; and where tentative conclusions are acceptable, an alpha-binary between 0.7 and 0.8 is tolerable (44).

For this study, inter-coder reliability was sufficient since Krippendorff's alpha-binary was 0.82 for the percentage of MI-consistent responses and 0.90 or higher for the open questions, closed questions and reflections, which were used to calculate the summary scores.

#### Sample Size

Based on a prior power analysis, 482 participants were required for this study, with 241 in each group, estimated on a small effect size [d = 0.25, α = 0.05, 1-ß = 0.80; (45)].

## Results

### Enrolment and Characteristics

#### Participants

**Figure 2:**
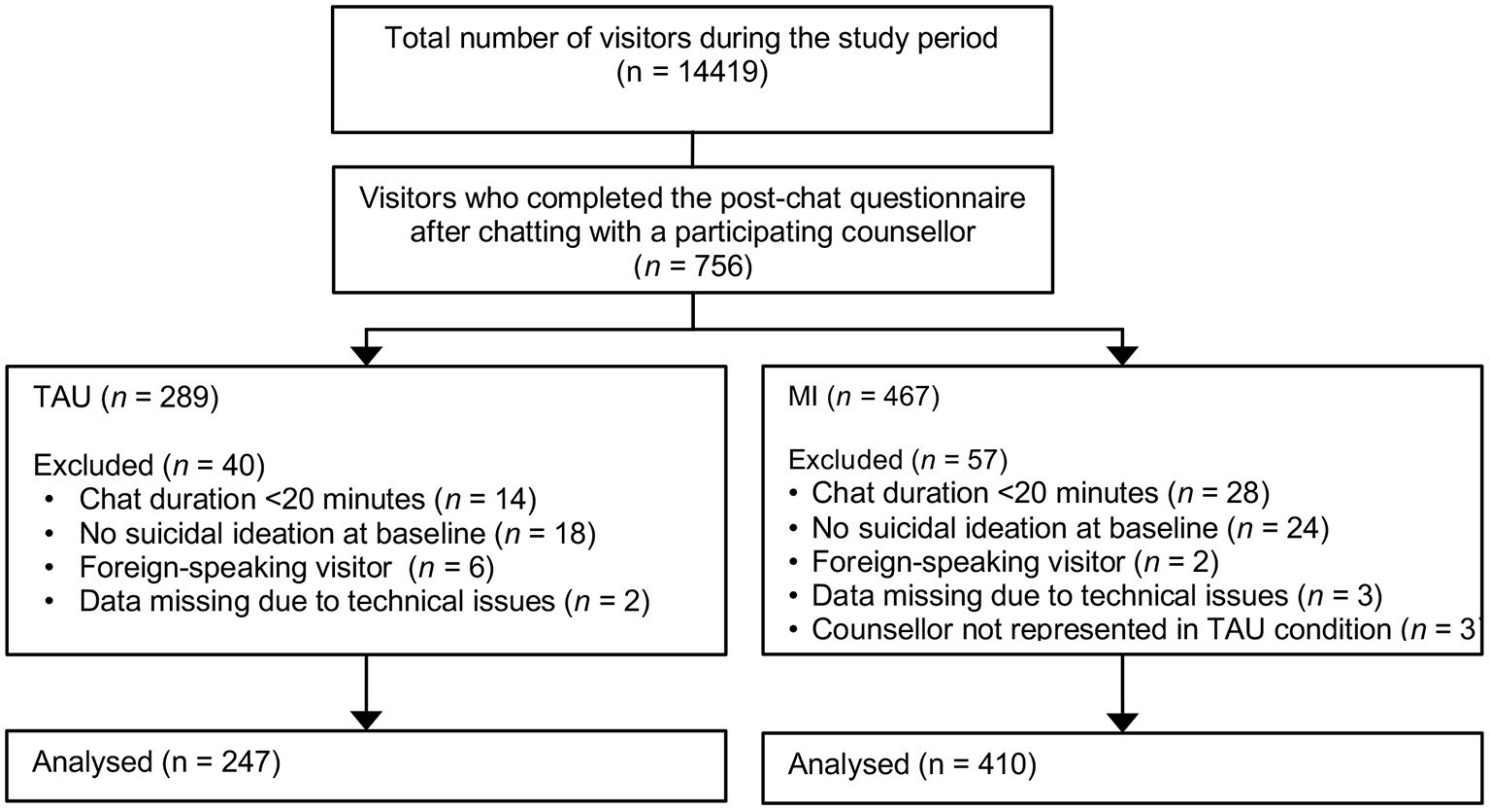
Participant flowchart.

Of the 14419 helpline visitors who visited the helpline during the data collection period, 756 were attended by one of the participating counselors and agreed to fill out the post-chat questionnaire. Figure 2 shows the number of participants who were in or excluded from the two intervention groups. While the last set of counselors were being trained, the first set had already carried out more conversations than expected, resulting in oversampling in the MI group.

The baseline characteristics of the participants in the two intervention groups are shown in Table 2. Seventy-nine per cent of the participants were female, and 80% were under the age of 35. There were no significant differences between the groups on any of the baseline characteristics.

**Table 2:** Baseline characteristics of the participants.

	**TAU**	**MI**	**Total**
**Demographic characteristics**			
Mean age (SD)	24.96 (12.76)	25.08 (10.93)	25.00 (11.76)
**Gender (%)**			
Male	16.2	20.0	19.29
Female	81.4	78.3	79.15
Other	2.4	1.7	1.56
**Mean scores on suicide risk factors (SD)**			
Suicidal ideation	5.41 (1.50)	5.29 (1.38)	5.11 (1.70)
Unbearable psychache	5.85 (1.44)	5.71 (1.41)	5.59 (1.61)
Defeat	5.81 (1.42)	5.71 (1.38)	5.53 (1.61)
Entrapment	6.32 (1.15)	6.37 (1.08)	6.19 (1.37)
Hopelessness	6.30 (1.09)	6.25 (1.03)	6.12 (1.34)
Perceived burdensomeness	5.90 (1.50)	5.73 (1.62)	5.61 (1.76)
Thwarted belongingness	5.17 (1.90)	5.10 (1.89)	4.96 (2.00)
Desire to live	2.86 (1.74)	2.71 (1.48)	2.92 (1.70)
Capability for suicide	4.65 (1.85)	4.63 (1.74)	4.44 (1.89)

#### Counselors

Fifty-seven counselors participated in the study, two of whom were excluded because they did not complete the entire training and two because they were not represented in both conditions. 28% of the counselors had a professional background, 13% were interns and 14% were volunteers. 11% of the counselors were male, 89% were female. Table 3 shows the characteristics of the counselors and chats stratified by condition.

**Table 3:** Characteristics of counselors and chats stratified by condition.

	**TAU**	**MI**
**Counselor characteristics**
Mean number of days worked at the helpline at the time of the chat (SD)	262.78 (359.92)	349.20 (242.41)
Average number of chats per counselor (SD)	4.66 (4.77)	7.74 (6.16)
**Chat characteristics**
Average duration in minutes (SD)	55.03 (17.27)	56.14 (19.53)

### Primary and Secondary Outcomes

**Figure 3:**
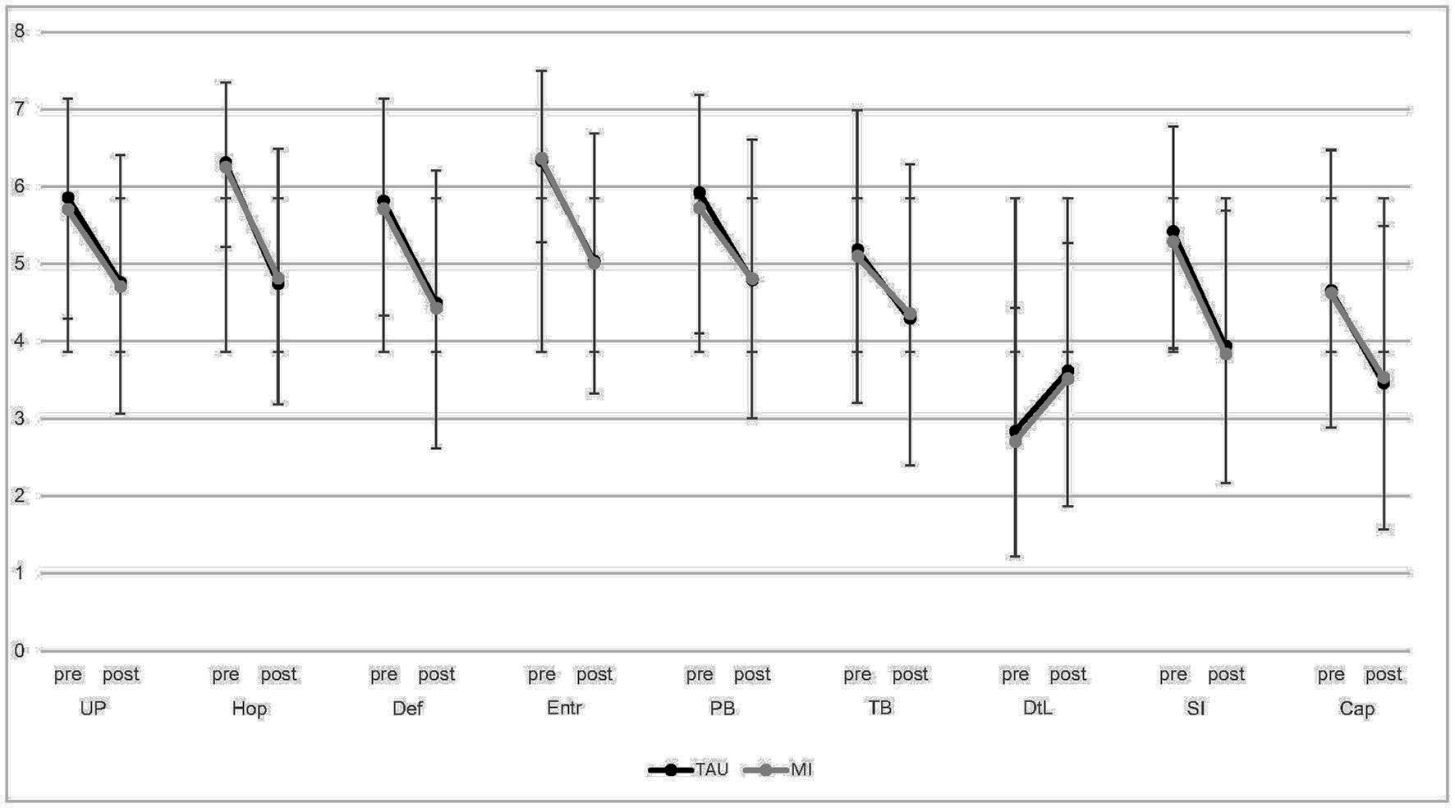
Means and standard deviations of pre- and post-chat scores for all suicide risk factors. SI, Suicidal ideation; UP, Unbearable psychache; Hop, Hopelessness; Def, Defeat; Entr, Entrapment; PB, Perceived burdensomeness; TB, Thwarted belongingness; DtL, Desire to live; Cap, Capability for suicide.

Figure 3 shows the means and standard deviations of all suicide risk factors before and after the chat. The mixed-model estimates can be found in Table 4. The effect of time was significant for all outcomes, which means that the scores on all the suicide risk factors had improved after the chat. The time × condition interaction was not significant for any of the outcomes, indicating that TAU and MI produced similar results. Adding working experience did not improve the performance of any of the models.

**Table 4:** Mixed-model estimates for all suicide risk factors in the full models.

**Variable**	**Time**			**Time x condition**		
	**ß**	**95% CI**	**p value**	**ß**	**95% CI**	**p value**
Suicidal ideation	−1.49	−1.70 to −1.28	<0.001^*^	0.03	−0.23 to 0.30	0.80
Unbearable psychache	−1.10	−1.29 to −0.90	<0.001^*^	0.10	−0.15 to 0.35	0.43
Hopelessness	−1.57	−1.77 to −1.36	<0.001^*^	0.13	−0.13 to 0.39	0.32
Defeat	−1.32	−1.53 to −1.10	<0.001^*^	0.04	−0.24 to 0.31	0.80
Entrapment	−1.29	−1.50 to −1.09	<0.001^*^	−0.06	−0.32 to 0.20	0.65
Perceived burdensomeness	−1.13	−1.34 to −0.93	<0.001^*^	0.22	−0.04 to 0.48	0.10
Thwarted belongingness	−0.89	−1.08 to −0.70	<0.001^*^	0.14	−0.09 to 0.39	0.23
Desire to live	0.08	0.57 to 0.99	<0.001^*^	0.03	−0.24 to 0.30	0.84
Capability for suicide	−1.20	−1.41 to −0.99	<0.001^*^	0.11	−0.15 to 0.38	0.41

* *Significant result (p < 0.05)*.

### Proficiency Level

Table 5 shows various indicators of MI proficiency. According to the benchmark criteria of both Moyers et al. (21) and Fischer (22), the counselors reached at least the beginner proficiency level on all summary scores, except for the reflection-to-question ratio. Furthermore, the percentage of change talk was far below the benchmark provided by Fischer (22).

**Table 5:** MI-SCOPE summary scores compared to benchmark criteria set forth by Moyers et al. (21) and Fischer (22).

	**Min**	**Max**	**Mean (sd)**	**Expert opinion benchmark (21)**	**Empirically derived** **benchmark (22)**
**Counselor summary scores**
% MI consistent	62.50	100.00	92.75 (8.79)	90.00^a^ 100.00^b^	75.00
% Open questions	11.11	100.00	61.08 (17.38)	50.00^a^ 70.00^b^	30.00
% Complex reflections	0.00	100.00	89.78 (19.23)	40.00^a^ 50.00^b^	30.00
Reflection-to-question ratio	0.00	2.44	0.47 (0.37)	1.00^a^ 2.00^b^	0.90
**Visitor summary scores**
% Change talk	0.00	100.00	40.37 (16.66)	NA	78.00

a *Beginner proficiency,*

b *Competency. NA, Not Applicable*.

## Discussion

The results of this study show that visitors of a chat-based suicide helpline showed improvement on several well-established suicide risk factors following their contact with the helpline, including suicidal ideation, unbearable psychache, entrapment, perceived burdensomeness and capability for suicide. We found no significant differences in any of the outcomes between the chat conversations handled by counselors using MI and those using TAU.

As for treatment proficiency, three of the four summary scores of the MI-SCOPE were above the thresholds provided by Fischer (22). Only the reflection-to-question ratio fell short of the benchmark, but this is arguably the least important of the summary scores as previous research did not show a significant relationship with the amount of change talk (22) or treatment outcomes (26). However, the visitor behavior scores showed that the visitors expressed less change talk and more sustain talk than expected. The percentage of change talk was 48%, far below the benchmark of 78%. This means that the counselors mainly used MI-consistent conversation techniques but were unable to strategically employ these techniques to elicit enough change talk and minimize the amount of sustain talk.

One explanation for the lack of change talk in this study might be that the counselors were insufficiently trained in the process of Evoking. Training in this study was relatively short, because resources at helplines are often sparse and staff turnover is high.

Furthermore, we reckoned it was more important to provide counselors with ongoing feedback and supervision after their initial training then to offer them an extensive workshop. We did not register how often counselors made use of these opportunities, however, and it is our impression that most counselors visited no more than one or two supervision sessions, which is probably not enough (46).

Second, it might be that there was simply not enough time for the process of Evoking, since this is one of the last processes of MI and chat is a rather slow medium. Also, the process of Engaging might take somewhat longer in a suicide prevention helpline then elsewhere, because it serves the extra purpose of letting visitors vent their emotions and get into their “window of tolerance”. This often requires a significant amount of sustain talk, at least if the target behavior is to refrain from committing suicide (and not to seek help, for which talking about your problems might be considered change talk). Current treatment fidelity measures do not assess during which processes change and sustain talk are expressed, however, so it is unclear to what extent this has influenced the ratio of change and sustain talk in this study.

At the same time, it might be argued that the benchmarks derived from other treatment settings might not be entirely appropriate for people in an emergency situation, in which it is to be expected that people express more negative and less positive thoughts and feelings.

### Strengths

The current study has a number of strengths. The effectiveness of chat-based suicide prevention helplines has rarely been investigated, even though such online services are increasingly being offered to vulnerable people worldwide. Furthermore, this is one of the first studies to investigate MI as a means of suicide prevention. The study was also sufficiently powered, used advanced statistical techniques and employed a control group, which is uncommon in this field. Furthermore, we assessed treatment integrity with an instrument measuring both the visitors' and counsellors' behavior, which enabled us to detect that the counselors had not elicited enough change talk. By using self-report measures, this study complements previous work which relied almost exclusively on counselor- and coder-rated measures. Another strength of this study was it's naturalistic setting.

### Limitations

Several limitations might have influenced the outcomes of this study.

First, visitors were not randomly assigned to a condition, and the post-chat questionnaire was voluntary. Only a very small portion of the people who visited the helpline during the study period filled out the post chat questionnaire, which might indicate selection bias: visitors who were satisfied with their chat might have been more inclined to fill out the questionnaire, resulting in an overestimation of the outcomes. The demographic characteristics of the sample were comparable to the general profile of people visiting the helpline, however, with the majority being female and younger than 30 years old.

Second, most of the outcomes reported in this study were measured using single-item self-report measures. There is evidence to suggest that such measures might not be as valid as longer measures (47). However, recent studies have shown that it is possible to measure suicide-related constructs using single items. For instance, De Beurs et al. (48) showed that all the items of the Entrapment Scale (36) performed equally well. Forkmann et al. (37) also found that several EMA items, including some of the items used in this study, correlated with longer, validated scales measuring the same constructs.

Furthermore, this study focused on the immediate and direct effects (as opposed to delayed or indirect effects) of the chat on visitors' suicide risk. For example, it might be that the counselor succeeded in motivating the visitor to try some sort of coping behavior or seek professional help but that the visitor had not actually taken these steps when the post-chat questionnaire was filled out. We did not assess the willingness of visitors to engage in such life-sustaining behaviors or conduct a follow-up assessment, although MI has previously been found to be effective in motivating people toward such behaviors (16, 17).

Since TAU and MI were not compared to a placebo or passive control group in this study, no conclusions can be drawn as to the effectiveness of suicide prevention helplines in general.

Finally, the two conditions were sampled in different seasons and suicide rates in The Netherlands are known to differ between seasons (49). This might have influenced the outcomes of this study, although there were no significant differences between the two groups at baseline and the main outcome of this study was suicidal ideation, not suicide. Also, there is no evidence to suggest that the outcomes of MI are susceptible to seasonal influences.

### Future Directions

We recommend that future studies include follow-up measurements or at least ask visitors about their intention to pursue alternatives to suicide at the end of the chat. To enable comparisons across helpline studies, we also recommend using more uniform, theoretically based outcomes, such as the well-established risk factors used in this study. Several short and validated measures are becoming available for this purpose (50).

It is important that future studies take measures to increase the number of visitors that fill out the post-chat questionnaire. For instance, the response rate might improve if the questionnaire is shorter, the counselor asks the visitor to fill out the questionnaire at the end of the chat and the questionnaire appears automatically when the chat is stopped.

We recommend that future studies on the effectiveness of MI measure treatment fidelity using a tool that not only measures the behavior of the counselor but also that of the client, such as the MISC (51) or the SCOPE (38). To date, most studies have used the MITI (52), which is relatively short and well validated, but it does not provide information on the amount of change and sustain talk expressed by the client. Had this study used the MITI, for instance, the treatment integrity indices would have indicated that the counselors used the right techniques, and the fact that they were unable to strategically deploy these techniques to elicit enough change talk would have gone unnoticed.

Eliciting change talk is the active ingredient of MI (26), and without it, MI and TAU would be largely identical. This might well explain why we found similar outcomes for the two groups and suggests that outcomes might improve if more change talk could be elicited, although it is far from uncommon for psychological interventions to produce equal results (53). It might be that the current results are all that can be expected from a single conversation with someone in such severe distress.

One way to help counselors elicit more change talk and minimize sustain talk is by improving their training. Future studies would do well to ensure that counselors attend at least four supervision sessions after their initial training and are provided with ongoing feedback, which seems to be especially important in learning MI (46). There are several validated proficiency measures for MI (40), and tools are already being developed to automatically measure treatment fidelity (54, 55). Such tools are especially interesting in relation to chat-based helplines, which could use them to provide counselors with rapid feedback on every chat they conduct, perhaps even while it is ongoing. Alternatively, counselors might be trained to assess the quality of their own sessions with a simplified treatment fidelity tool. There are digital simulators that might help improve and assess counsellors' proficiency in a cost-effective manner and tailor additional training to their needs.

Second, counselors should be encouraged to keep the process of Engaging as concise as possible, especially if the conversation is conducted over chat, since visitors often share a great deal of sustain talk during this process. Furthermore, if engaging takes longer than necessary, less time will be devoted to the process of evoking, in which change talk is to be elicited. Off course this should not be done at the expense of establishing a good working alliance, which is an important predictor of treatment outcome in most if not all psychological interventions (56), including MI (57).

Finally, helplines might be more effective if they offer more extensive forms of help. For example, they might direct users to a website with self-help resources after the chat, follow-up on users in the days or weeks after their contact with the helpline or offer them online therapy, such as brief cognitive behavioral therapy for suicide prevention (58).

## Conclusions

This non-randomized controlled trial showed that counselors of a chat-based suicide prevention helpline can learn to use MI-consistent conversation techniques after a relatively short period of training. Training counselors in MI did not lead to greater reductions in self-reported suicide risk factors, however, probably because the counselors could not strategically deploy these techniques to elicit enough change talk. However, the effectiveness of MI would have likely improved if the counselors were able to elicit more change talk and there was an active community of clinicians and researchers working with MI. Thus, helplines working with MI can benefit from knowledge and resources created elsewhere. While MI can already be used on suicide prevention helplines, there is ample room for improvement.

## Data Availability Statement

The raw data supporting the conclusions of this article will be made available by the authors, without undue reservation.

## Ethics Statement

The studies involving human participants were reviewed and approved by VU University Medical Centre in Amsterdam. Written informed consent from the participants' legal guardian/next of kin was not required to participate in this study in accordance with the national legislation and the institutional requirements.

## Author Contributions

WJ designed the study, prepared the data, performed some of the statistical analyses, supervised coding of the chats, and wrote the article. JR prepared the data, performed most of the analyses, and coded half of the chats. YL helped prepare the data and coded half of the chats. SM designed the study, supervised analyses, interpretation of the results, and revised the article. WB supervised analyses, interpretation of the results, and revised the article. All authors contributed to the article and approved the submitted version.

## Funding

This study was funded by 113 Suicide Prevention.

## Conflict of Interest

The authors declare that the research was conducted in the absence of any commercial or financial relationships that could be construed as a potential conflict of interest.

## Publisher's Note

All claims expressed in this article are solely those of the authors and do not necessarily represent those of their affiliated organizations, or those of the publisher, the editors and the reviewers. Any product that may be evaluated in this article, or claim that may be made by its manufacturer, is not guaranteed or endorsed by the publisher.
