# Supplementary material for: Can Outcomes of a Chat-Based Suicide Prevention Helpline Be Improved by Training Counselors in Motivational Interviewing? A Non-randomized Controlled Trial
Source: Front Digit Health. 2022 Jun 21;4:871841. doi: 10.3389/fdgth.2022.871841 (PMC9253377; doi:10.3389/fdgth.2022.871841)
Supplement: Supplementary file 1 [file Data_Sheet_1.pdf]

Appendix A – Content of MI Training

Subject	Training method	% of training devoted to subject
Spirit & rationale	Didactic instruction	1
Processes	Didactic instruction	1
Conversation techniques	Didactic instruction	6
	Experiential exercise	2
	Role-play	3
Total – basic principles & techniques		13
Engaging	Didactic instruction	3
	Experiential exercise	1
	Generate own example phrases	2
	Role-play	15
Total – Engaging		21
Focussing	Didactic instruction	7
	Experiential exercise	2
	Generate own example phrases	2
	Role-play	9
Total – Focussing		20
Evoking	Didactic instruction	8
	Experiential exercise	1
	Generate own example phrases	4
	Role-play	17
Total – Evoking		30
Planning	Didactic instruction	4
	Generate own example phrases	2
	Role-play	10
Total – Planning		16

Appendix B – Mean pre- and postchat scores with SDs

	suicidal ideation		hopelessness		entrapment		perceived burdensomeness		desire to live		capability for suicide		thwarted belongingness		defeat		Unbearable psychache	
	pre	post	pre	post	pre	post	pre	post	pre	post	pre	post	pre	post	pre	post	pre	post
TAU	5.425 (1.49)	3.939 (1.85)	6.312 (1.09)	4.745 (1.67)	6.332 (1.13)	5.04 (1.67)	5.927 (1.46)	4.794 (1.80)	2.838 (1.73)	3.619 (1.76)	4.664 (1.85)	3.466 (1.96)	5.186 (1.89)	4.296 (1.93)	5.814 (1.43)	4.498 (1.78)	5.862 (1.43)	4.765 (1.7)
MI	5.29 (1.38)	3.839 (1.67)	6.254 (1.03)	4.82 (1.63)	6.366 (1.08)	5.015 (1.69)	5.727 (1.62)	4.81 (1.8)	2.707 (1.48)	3.517 (1.65)	4.627 (1.74)	3.539 (1.97)	5.102 (1.89)	4.359 (1.96)	5.712 (1.38)	4.432 (1.81(	5.705 (1.41)	4.707 (1,64)

Table 1 mean pre- and postchat scores with standard deviations for both treatment groups

Appendix C – Mixed model estimates for the models with working experience added as a random effect

Variable	Time			Time x condition		
	ß	95% CI	p value	ß	95% CI	p value
Suicidal ideation	-1.49	-1.70 to -1.28	<.001*	0.03	-0.23 to -0.30	0.80
Unbearable psychache	-1.10	-1.29 to -0.90	<.001*	0.10	-0.15 to 0.35	0.43
Hopelessness	-1.57	-1.77 to -1.36	<.001*	0.13	-0.13 to 0.39	0.32
Defeat	-1.32	-1.54 to -1.10	<.001*	0.04	-0.24 to 0.31	0.80
Entrapment	-1.29	-1.50 to -1.09	<.001*	-0.06	-0.32 to 0.20	0.65
Perceived burdensomeness	-1.13	-1.34 to -0.93	<.001*	0.22	-0.04 to 0.48	0,10
Thwarted belongingness	0.89	-1.08 to -0.70	<.001*	0.15	-0.09 to 0.39	0.23
Desire to live	0.08	0.57 to 0.99	<.001*	0.03	-0.24 to 0.30	0.84
Capability for suicide	-1.20	-1.41 to -0.99	<.001*	0.11	-0.15 to 0.38	0.41

* significant result (p < .05)
